# A Mathematical Approach to Consider Solid Compressibility in the Compression of Pharmaceutical Powders

**DOI:** 10.3390/pharmaceutics11030121

**Published:** 2019-03-15

**Authors:** Isabell Wünsch, Jan Henrik Finke, Edgar John, Michael Juhnke, Arno Kwade

**Affiliations:** 1 Institute for Particle Technology, Technische Universität Braunschweig, Volkmaroder Straße 5, Braunschweig 38104, Germany; jfinke@tu-braunschweig.de (J.H.F.); a.kwade@tu-braunschweig.de (A.K.); 2 Center of Pharmaceutical Engineering (PVZ), Technische Universität Braunschweig, Braunschweig 38106, Germany; 3 Novartis Pharma AG, Basel 4002, Switzerland; edgar.john@novartis.com (E.J.); michael.juhnke@novartis.com (M.J.)

**Keywords:** powder compression, tableting, compression equations, solid compressibility, in-die compression analysis

## Abstract

In-die compression analysis is an effective method for the characterization of powder compressibility. However, physically unreasonable apparent solid fractions above one or apparent in-die porosities below zero are often calculated for higher compression stresses. One important reason for this is the neglect of solid compressibility and hence the assumption of a constant solid density. In this work, the solid compressibility of four pharmaceutical powders with different deformation behaviour is characterized using mercury porosimetry. The derived bulk moduli are applied for the calculation of in-die porosities. The change of in-die porosity due to the consideration of solid compressibility is for instance up to 4% for microcrystalline cellulose at a compression stress of 400 MPa and thus cannot be neglected for the calculation of in-die porosities. However, solid compressibility and further uncertainties from, for example the measured solid density and from the displacement sensors, are difficult or only partially accessible. Therefore, a mathematic term for the calculation of physically reasonable in-die porosities is introduced. This term can be used for the extension of common mathematical models, such as the models of Heckel and of Cooper & Eaton. Additionally, an extended in-die compression function is introduced to precisely describe the entire range of in-die porosity curves and to enable the successful differentiation and quantification of the compression behaviour of the investigated pharmaceutical powders.

## 1. Introduction

Powder compaction is an important production process in diverse industries, such as food, ceramic and pharmaceutical industry. The prediction of structural and mechanical properties of tablets based on raw material properties and process parameters is still difficult or rather impossible, although the powder compaction process was the object of numerous scientific investigations. The main reason for the incomplete predictability is certainly the still limited process understanding due to the complexity of the powder compaction process. This complexity can be attributed to various influencing parameters [1,2,3,4,5], such as the deformation behaviour, the particle size and shape and the compression stress on the one hand and to different acting micro-processes [6,7,8,9,10], such as particle rearrangement, elastic and plastic deformation of single particles and particle fragmentation on the other hand. These micro-processes do not take place sequentially but occur simultaneously. The contribution of the single mechanisms depends on material properties and applied process parameters. Until today, the individual quantitative characterization of each mechanism is challenging. The deformation behaviour also affects the number of generated, inter-particulate bonds as well as the bonding forces and thus the resulting structural and mechanical properties of the tablet [11,12]. The missing predictability causes the need for a systematic and comprehensive characterization of the compression and compaction behaviour of raw materials as prerequisite for a rationally based formulation and process development.

This study is focused on the characterization of powder compressibility. The compressibility is defined as the relationship between solid fraction/porosity and compression stress [8]. Several mathematical models were developed for the description of the compression curves and the derivation of specific compression parameters. An overview is given by Kawakita and Lüdde [13] and Celik [14], for example. The main requirement for a suitable process function is the ability to describe the compression curves of related materials precisely and robustly. The simplest functions are two-parametric equations, such as the common models of Heckel [15] and of Kawakita and Lüdde [13]. These functions only depict a limited part of the compression curve. In contrast, four-parametric models enable the description of the entire compression curve. A common four-parametric process function is the model of Cooper & Eaton [16].

The most common method for the determination of compression curves is the out-of-die analysis and the resulting compressibility is named out-of-die compressibility. In this case, tablets are produced by applying different compression stresses and related tablet solid fraction/porosity is determined after storage of the tablets at defined conditions for a period of time. However, this method is only applicable with limitations when tablet defects, like lamination or capping, occur. Instrumented tablet presses, equipped with force and displacement sensors, enable the determination of the compressibility during compression (in-die compressibility). In this case, solid fraction/porosity is calculated based on the measured force-displacement curve, taking the tablet mass and its solid density into account. The main advantage of in-die analysis is that, in the best case, the performance of only one compression process is sufficient for the comprehensive characterization of powder compressibility. Furthermore, in-die compressibility can be determined even if tablet defects occur. However, in-die compressibility is typically shifted to higher solid fractions/lower porosities compared to out-of-die compressibility, which lead to different specific compression parameters [17,18,19,20]. The main reason for the observed differences is the inclusion of elastic deformation in the case of in-die analysis, while out-of-die analysis does not take elastic deformation into account as compressed powders relax during and after unloading. Additionally, physically unreasonable apparent solid fractions above one/apparent porosities below zero are observed for in-die analysis above certain compression stress levels [21,22]. For this reason, in-die data presented in literature are often limited to lower compression stresses that may affect derived specific compression parameters [3,19,23]. One reason for the occurrence of physically unreasonable values is the assumption of a constant solid density [19,21]. The molecular lattice inside the individual particles deforms elastically during compression as well and this leads to a decrease of specific solid volume upon compression [21,24,25,26]. Consequently, solid density increases with rising compression stress, especially for organic substances. This phenomenon is referred to as solid compressibility. As one example, Boldyreva showed for paracetamol a decrease in specific solid volume of approximately 3% at hydrostatic compression of 400 MPa [24]. Although different researchers mention the influence of solid compressibility as an important influencing factor on in-die analysis, this phenomenon is scarcely considered. Sun and Grant introduced a term for the correction of in-die porosity in dependence of the Young’s modulus of the material [17]. They showed that the deviation between the yield strength derived by in-die analysis and out-of-die analysis increases with decreasing Young’s modulus. Sun and Grant did not differentiate between elastic deformation of the bulk and the single particles and solid compressibility. Additionally, Sun and Grant did not consider the stress-dependency of solid density. The common neglect of the stress-dependency of solid density might be explained by the inaccessibility of this phenomenon during powder compression. The volume change of single crystals can be measured using crystallographic methods, however, such measurements need to be performed by X-ray diffraction in sufficiently resilient dies. Another method for the determination of solid compressibility is mercury porosimetry provided that the single particles are completely enclosed by mercury and that all pores are completely filled with mercury [27,28].

Further reasons for the occurrence of physically unreasonable solid fractions/porosities are uncertainties of the compressed mass, of the measured solid density and from the force and displacement sensors [19,29,30,31]. Another influencing parameter could be the die diameter, which is assumed to be constant. However, the expansion of the die was not considered yet. These challenges are the reason for the still more frequent use of out-of-die compressibility analysis, although in-die analysis is material and time-saving. Katz et al. introduced a new empirical approach to use the advantages of both methods [18,20]. They calculated out-of-die compressibility based on in-die compressibility under consideration of elastic and viscoelastic recovery. The performance of only two compression experiments, one at relatively low compression stress and one with maximum compression stress, are sufficient for this approach. This method is consequently not applicable if tablet defects occur. However, the characterization of the compression behaviour of materials which tend to form defective tablets is important for formulation and process development. It can be concluded that a method for the calculation of physically reasonable in-die porosities/solid fractions is needed for establishing in-die analysis for formulation and process development.

In this study, the influence of solid compressibility and of die expansion on in-die compression analysis is investigated for pharmaceutical materials with considerably different deformation behaviour. Additionally, a mathematical term for the pragmatic consideration of solid compressibility during in-die compression analysis and hence, for the calculation of physically reasonable in-die porosities is introduced. Its applicability combined with the common compression models of Heckel and of Cooper & Eaton, as well as an extended in-die compression function are discussed.

## 2. Theory

Various mathematical models for the description of powder compressibility exist in literature. Common models in industrial pharmacy are the models of Heckel [15] and of Cooper & Eaton [16], which are used in this study.

### 2.1. Model of Heckel

The model of Heckel [15] was originally developed for the compaction of metal powders with a mainly ductile deformation behaviour. Heckel assumed that the porosity decrease during powder compaction follows a first order kinetic:

(1) $$ln\frac{1}{\varepsilon }=k\cdot \sigma_{ax}+A$$

where *ε* is the porosity, *σ_ax_* is the axial compression stress and *k* and *A* are constants. Heckel found that the constant k correlates with the reciprocal value of yield strength *σ*0 for metals and is thus a measure of the plasticity of a material [32]. Hersey and Rees introduced the term mean yield pressure *P*y for this correlation [33]:

(2) $$\;k=\frac{1}{3\sigma_{0}}=\frac{1}{P_{y}}$$

### 2.2. Model of Cooper & Eaton

Cooper and Eaton describe the compression process of ceramic powders by two steps: the filling of inter-particulate pores in the size range similar to the particle size and the filling of smaller pores [16]. They hypothesize particle rearrangement as the main deformation mechanism responsible for the first step and plastic deformation and particle fragmentation as the main deformation mechanisms of the second step. Cooper and Eaton consider the volume change in dependence on compression stress and used two exponential terms for the description of both mechanisms:

(3) $$V^{*}=\frac{V_{0}-V}{V_{0}-V_{\infty }}=a_{1}\;exp\left(-\frac{k_{1}}{\sigma_{ax}}\right)+a_{2}\;exp\left(-\frac{k_{2}}{\sigma_{ax}}\right)$$

where V* is the relative volume change, *V*_0_ is the initial volume, *V*_∞_ is the powder volume at infinite compression stress and *a*_1_, *a*_2_, *k*_1_ and *k*_2_ are constants, with the boundary condition of the sum of *a*_1_ and *a*_2_ equalling one. *a*_1_ and *a*_2_ are the fractions of theoretically possible compression and describe the maximum possible volume reduction attributed to each respective mechanism. *k*_1_ and *k*_2_ are characteristic compression values, which indicate the stress range, in which the particular mechanism dominates the volume change. In this study, the initial specific volume *V*_0_ is calculated using the data of the first measurement point of the force-displacement curve. The specific solid volume determined by helium pycnometry is used as specific volume at infinite compression stress *V*_∞_.

## 3. Materials

Microcrystalline cellulose (MCC, Vivapur^®^ 102, JRS Pharma, Rosenberg, Germany), anhydrous lactose (Lac, Lactose Anhydrous NF DT, Sheffield Bio Science, Norwich, NY, USA), anhydrous dicalcium phosphate (DCPA, DI-CAFOS^®^ A150, Chemische Fabrik Budenheim, Budenheim, Germany) and paracetamol (Para, Novartis Pharma AG, Basel, Switzerland) were selected as pharmaceutical materials while magnesium stearate (Faci, Carasco, Italy) was used as lubricant.

**Figure 1:**
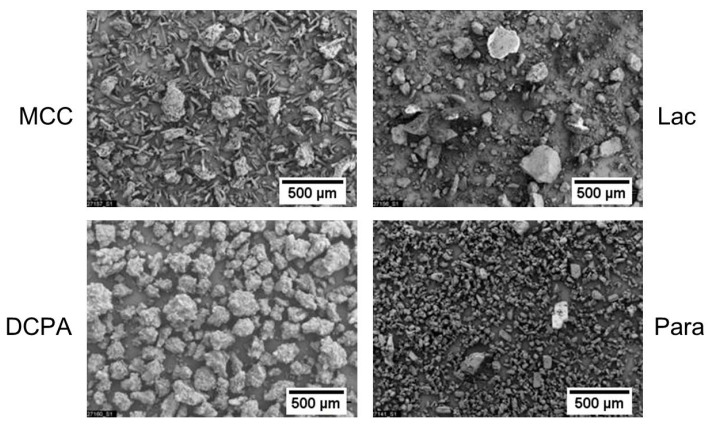
Particle images of the pharmaceutical powders MCC, Lac, DCPA and Para determined by scanning electron microscopy.

The characteristic particle sizes of MCC, Lac and DCPA are in a comparable range but differ in width, while Para is clearly finer (Table 1). The particle shape of all model materials is irregular (Figure 1). MCC mainly consists of elongated primary particles and of approximately spherical agglomerates or aggregates. The primary particles of Para are approximately rectangular. In contrast, Lac and DCPA consist of irregularly shaped aggregates and agglomerates.

## 4. Methods

### 4.1. Powder Characterization

Solid density of the powders was determined using the helium pycnometer ULTRAPYC 1200 (Quantachrome Instruments, Boynton Beach, FL, USA). The powders were dried under vacuum for 24 h before the measurement. Double measurements with 10 measurement points each were performed and the mean values (Table 1) were used for the calculation of apparent in-die porosities. Additionally, the true density of paracetamol was determined using the X-ray powder diffraction (Bruker D8 Advance, Billerica, MA, USA). Three measurements were performed at room temperature and Rietveld refinement was applied. Furthermore, particle size analysis was performed using the laser light diffraction instrument HELOS (Sympatec, Clausthal-Zellerfeld, Germany) in combination with the dry dispersion unit RODOS for the excipients and the wet dispersion unit CUVETTE for Para.

### 4.2. Determination of the Bulk Modulus

The solid compressibility of the powders was characterized using the mercury porosimeter PoreMaster^®^ GT60 (Quantachrome Instruments, Boynton Beach, FL, USA). The penetrometer with a volume of 0.5 cm^3^ was filled with an appropriate amount of powder. The low pressure operation (up to 344 kPa) was used for the filling of the penetrometer with mercury and is not considered for the determination of solid compressibility because of the probably incomplete enclosure of the single particles by mercury. The maximum applied pressure of the high pressure operation was 414 MPa. Four samples per material were characterized and high pressure operation was repeated five times per sample to ensure elastic deformation as the reason for volume changes. Before the analysis, several blank measurements were performed for the consideration of the volume change due to the elastic deformation of the penetrometer and of mercury.

The elastic compressibility of an isotropic material under hydrostatic pressure can be described by the bulk modulus K:

(4) $$K=-V_{0}\frac{dp}{dV}$$

where *V*_0_ is the volume at ambient pressure and *p* is the hydrostatic pressure and d*p* can be replaced by *p*−*p*_0_ and d*V* by *V*−*V*_0_. *p*_0_ is the atmospheric pressure and can be neglected for powder compression. This results in

(5) $$\frac{V_{S}\left(p\right)}{V_{S,0}}=1-\frac{p}{K}$$

where *V*_S_ is the specific solid volume dependent on the hydrostatic pressure and *V*_S,0_ is the specific solid volume at atmospheric pressure. The bulk modulus is hence the reciprocal of the slope of the measured volume change during mercury intrusion and was determined by linear regression using the software Excel 2010with the solver add-in (Microsoft, Redmond, WA, USA). It has to be noted that not the whole pressure range is considered because of non-linearity at lower pressure (frequently < 150 MPa), which indicates the presence of still unfilled pores and the still insufficient enclosure of the single particles by mercury. The bulk modulus was determined for all performed measurements and the average value was calculated per material.

### 4.3. Tableting

Compaction experiments were performed using the compaction simulator Styl’One Evolution (MEDEL’PHARM, Beynost, France), which is equipped with force and displacement sensors. The die and the punches were manually pre-lubricated with magnesium stearate. 450 mg powder of each MCC, Lac and Para as well as 900 mg of DCPA were compressed (*n* = 6) with a maximum compression stress of 400 MPa using 11.28 mm flat-faced, round punches. The compression profile of a camshaft was simulated with a maximum compression velocity of approximately 20 mm/s. The weight of the tablets was measured after compaction and in-die analysis was performed considering machine deformation. Additionally, experiments using an instrumented die (11.28 mm Euro B) (MEDEL’PHARM, Beynost, France) were performed for the determination of the radial die wall stress with a maximum compression stress of 300 MPa. The stress ratio, which is defined as the ratio between radial and axial stress, is calculated based on the maximal axial and radial stresses.

The tablets were stored under constant ambient conditions for at least 24 h. Afterwards, the weight of the tablets (*n* = 20) was measured and geometrical dimensions were determined using the tablet tester MultiTest 50 FT (Dr. Schleuniger, Aesch, Switzerland). The data were used for the calculation of out-of-die tablet porosity.

### 4.4. Application of the Compression Equations

The fitting of the models to experimental data was performed using regression by minimization the sum of squared errors. The software Excel 2010 (Microsoft, Redmond, WA, USA) was used for the fitting of the models of Heckel and of Cooper & Eaton. The fitting of the extended in-die compression function was performed using MATLAB (Version: R2015b, MathWorks, Natick, MA, USA). Additionally, out-of-die Heckel analysis was performed by application of the model of Heckel to the out-of-die porosities between 200 MPa and 400 MPa.

### 4.5. Estimation of Radial Die Expansion

The elastic expansion of the die in radial direction during powder compression is estimated under assumption of rotationally symmetric and height-independent stress conditions inside the die wall based on Hook’s law [34]:

(6) $$\varepsilon_{rad}=\frac{\sigma_{rad}}{E}-\frac{\nu \cdot \sigma_{tan}}{E}$$

where ε_rad_ is the elastic strain in radial direction, σ_rad_ is the stress in radial direction, σ_tan_ is the elastic stress in tangential direction, E is the Young’s modulus and ν the Poisson’s ratio. The radial stress was measured using an instrumented die, while the tangential stress for a cylinder with a thick wall (r_a_/r_i_ ≥ 1.2 according to DIN 2413) can be estimated as follows [35]:

(7) $$\sigma_{tan}=\;\frac{r_{i}^{2}\cdot p}{r_{a}^{2}-r_{i}^{2}}\left(1+\frac{r_{a}^{2}}{r^{2}}\right)$$

where *r*_i_ is the inner radius, *r*_a_ is the outer radius and *p* is the inner pressure acting on the die wall. The estimation of die expansion was performed for Euro D compression tools with an inner radius of 5.64 mm and an outer radius of 19.05 mm using a Young’s modulus of 210 GPa and a Poisson’s ratio of 0.28, which are typical values of stainless steel. The influence of the die holder and the groove are neglected.

## 5. Results and Discussion

### 5.1. Compression Behavior of the Model Materials

**Figure 2:**
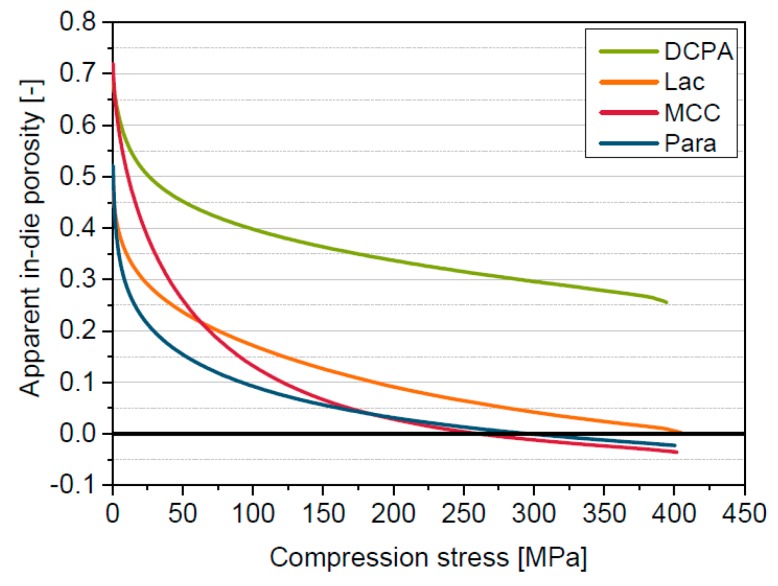
Apparent in-die compressibility of MCC, Lac, DCPA and Para.

The in-die compression curves of the four materials differ considerably (Figure 2). The slope of the curve of Para is the highest at the beginning of the compression process (below approximately 50 MPa) indicating the lowest resistance against compression. In contrast, DCPA shows the overall highest apparent in-die porosity and the lowest porosity decrease. Hence, the effective compression of DCPA is the lowest. MCC and Lac show behaviour in between that of the aforementioned. Furthermore, it has to be noted that physically not reasonable negative apparent in-die porosities are reached for Para and MCC at compression stresses above 200 MPa. The reasons for the occurrence of negative in-die porosities are discussed in the further sections.

### 5.2. Reasons for Apparent in-Die Porosities Below Zero

**Figure 3:**
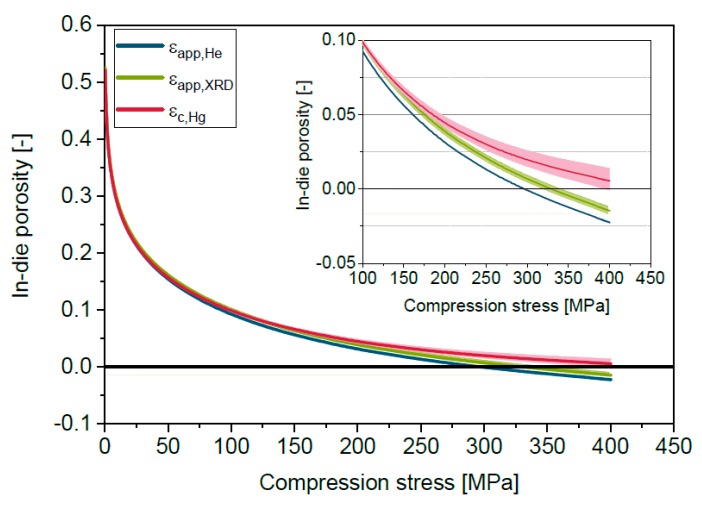
Apparent in-die compressibility of Para calculated using the solid density determined by helium pycnometry ε_app,He_ and by X-ray diffraction ε_app,XRD_ and correction of the apparent in-die compressibility ε_c,Hg_ with the bulk modulus determined by mercury porosimetry. The filled areas mark the 95% confidence interval regarding density ε_app,He_ and ε_app,XRD_ or rather the bulk modulus ε_c,Hg._

A fundamental parameter for the calculation of porosities is the solid density, which is commonly determined by helium pycnometry. Helium pycnometry is applicable regardless of the degree of crystallinity in contrast to X-ray diffraction. However, variations of the solid density determined by helium pycnometry are often reported due to the influence of material properties and measurement conditions [31,36,37]. The solid density of Para determined by helium pycnometry is slightly lower (1.2863 ± 0.001 g/cm^3^) compared to the true density determined by X-ray diffraction (1.2936 ± 0.0027 g/cm^3^). The slightly lower density determined by helium pycnometry might be explained by the consideration of closed intra-particulate pores and flaws, while these are not taken into account by evaluation of X-ray diffraction. The applied high stress during powder compression causes particle deformation and fragmentation, which may lead to the disappearance of closed pores and flaws. Therefore, the usage of the true density determined by X-ray diffraction seems to be useful for powder compression. Since often the solid density determined by helium pycnometry is used, it is necessary to consider the influence of the differences between solid density and true density on the calculation of in-die porosities. The lower solid density determined by helium pycnometry causes a slight shift of the apparent in-die porosity ε_app,He_ curve of Para to slightly lower porosities compared to the curve calculated using the true density determined by X-ray diffraction (ε_app,XRD_) (Figure 3). However, in both cases physically not reasonable negative apparent in-die porosities are reached at high compression stresses. The apparent in-die porosities are calculated based on the assumption of a constant solid density during powder compression, although solid compressibility causes the increase of solid density with rising compression stress [19,21], especially for organic materials. Therefore, the bulk modulus measured by mercury porosimetry (Table 2) is used for the calculation of a stress-dependent specific solid volume according to Equation (5) based on the specific solid volume determined by helium pycnometry. The resulting in-die compression curve ε_c,Hg_ is clearly shifted to the positive porosity range, especially at compression stresses above 200 MPa, as Figure 3 shows exemplarily for Para. The same trend is found for MCC and Lac (Table 2). The bulk modulus of the inorganic DCPA could not be determined by mercury porosimetry because of reaching the detection limit. The bulk modulus of DCPA is hence to be expected clearly higher compared to the three organic materials. The bulk modulus of DCPA could be alternatively calculated using the Young’s modulus and Poisson’s ratio. Beam bending of compactates or double compaction with a fully instrumented compaction simulator can be used for the determination of Young’s modulus and Poisson’s ratio of compactates with defined porosities [38,39,40]. Extrapolation to zero porosity can then be applied for the derivation of properties of the solid, which are needed for the calculation of the bulk modulus. Additionally, high-pressure diffraction studies of single particles could be performed [24].

The minimal in-die porosities change by approximately 3% for Para, by 4% for MCC and by 1.7% for Lac. Accordingly, solid compressibility has a significant effect during powder compression and should be considered for the calculation of in-die porosities. The porosity change of DCPA is expected to be considerably lower than 1.7%. However, the transferability of the bulk modulus determined by hydrostatic compression to uniaxial compression might be limited. The stresses acting on the single particles during uniaxial powder compression are unknown and, additionally, powder compression is anisotropic and dynamic, whereas material bulk moduli are generally measured at defined pressures and thermodynamic equilibrium. In addition, the stress directed to the die wall is complex and depends on material and process parameters [41,42]. Additionally, the accuracy of mercury porosimetry for the determination of the bulk modulus and uncertainties of the measured solid density by helium pycnometry has to be considered.

**Figure 4:**
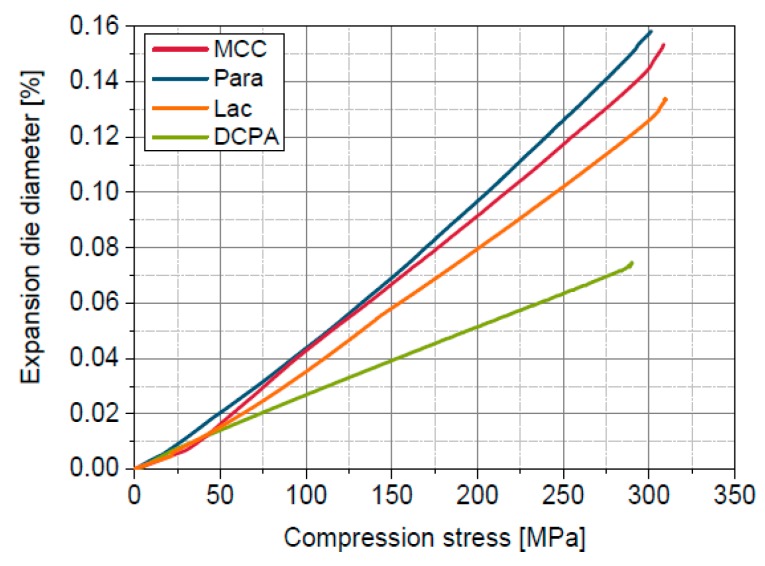
Expansion of the die diameter in dependence on the applied compression stress for Para, MCC, Lac and DCPA.

An additional influencing factor could be the elastic expansion of the die, which is dependent on the radial stress acting on the die wall. The radial stress during powder compression is dependent on the stress ratio between radial and axial compression stress, which is in turn determined by the axial compression stress and the used material in combination with its deformation behaviour (Table 3). The stress ratio in the range from approximately 0.5 MPa to 400 MPa is the highest for MCC and Para because of the ductile and elastic behaviour of these materials [43] leading to the largest die expansion (Figure 4). The stress ratio of Lac is in the medium range, while the lowest stress ratio was found for DCPA (Table 3), which correlates well with the findings of Abdel-Hamid and Betz [43]. Accordingly, the expansion of the die, calculated using Equations (6) and (7), is lower for Lac and DCPA. For the direct comparison of the influence of the expansion of the die diameter with the effect of solid compressibility, the die expansion coefficient was introduced and defined as the reciprocal of the slope of the die expansion curves (Figure 4). The die expansion coefficients are considerably larger than the bulk moduli (Table 2 and Table 3). Die expansion leads to small changes of the calculated in-die porosities, which are additionally recognizable by the estimated difference of porosity at maximum compression stress of 400 MPa, comparing in-die porosity determined with die expansion to in-die porosity determined without die expansion (Table 3). The influence of die expansion is therefore negligible for the calculation of in-die porosities.

Other influencing parameters for the calculation of apparent in-die porosity could be mass variations and the accuracy of the displacement sensors, as other studies showed [30,31]. The experimental determination of all uncertainties for the calculation of in-die porosities is fundamentally feasible but highly expensive. However, in-die compression analysis is an advantageous method for the characterization of powder compressibility and can be applied as well for materials, which tend to form defective tablets. For this reason, a pragmatic mathematical approach for consideration of the physical effect of solid compressibility and other less important mechanisms is introduced.

### 5.3. Introduction of the Solid Compressibility Term

Equation (5) is valid for the volume change under hydrostatic compression. For uniaxial powder compression, the hydrostatic pressure can be approximated as follows [44]:

(8) $$p=\frac{\sigma_{ax}+2\cdot \sigma_{rad}}{3}$$

where *σ*_ax_ is the stress in axial direction and *σ*_rad_ is the stress in radial direction. The axial and radial stresses acting on the single particles during uniaxial powder compression are unknown and cannot be measured yet due to the highly complex stress state inside the powder. In reality, a stress distribution exists due to the bulk structure. Usually, instrumented tablet presses enable only the measurement of the overall axial stress acting on the powder. The overall radial stress can be determined using an instrumented die. However, the usage of instrumented dies is not common because of difficulties in design, operation and data evaluation [45]. The estimation of the stresses acting on the single particles based on the measured data is not possible, yet, because of the complexity of influencing parameters. Therefore, the hydrostatic pressure is approximated by the overall axial compression stress acting on the powder. Furthermore, investigations of the compressibility of single crystals by hydrostatic compression with high pressures up to several GPa show the decrease of specific solid volume with rising hydrostatic pressure according to the Murnaghan equation [46]. This equation implies a stress dependency of the bulk modulus. The stress range applied for tableting is considerably lower compared to the applied stresses for single crystal analysis. Thus, a constant bulk modulus is assumed within the context of this investigation. The bulk modulus K of Equation (5) is replaced by the elastic compressibility factor *C*, which mainly comprises the influence of solid compressibility. According to these assumptions Equation (5) is modified as follows:

(9) $$V_{S}\left(\sigma_{ax}\right)=V_{S,0}\cdot \left(1-\frac{\sigma_{ax}}{C}\right)$$

where *σ*_ax_ is the overall axial compression stress acting on the powder and *C* is here referred to as elastic compressibility factor. The in-die porosity under consideration of solid compressibility *ε*_c_ is given by

(10) $$\varepsilon_{c}\left(\sigma_{ax}\right)=1-\frac{V_{s}\left(\sigma_{ax}\right)}{V_{b}\left(\sigma_{ax}\right)}$$

Inserting Equation (9) in Equation (10) leads to the following mathematical relation for the in-die porosity under consideration of solid compressibility *ε*_c_

(11) $$\varepsilon_{c}\left(\sigma_{ax}\right)=1-\frac{V_{S,0}\cdot \left(1-\frac{\sigma_{ax}}{C}\right)}{V_{b}\left(\sigma_{ax}\right)}=1-\frac{V_{s,0}}{V_{b}\left(\sigma_{ax}\right)}+\frac{V_{S,0}\cdot \sigma_{ax}}{V_{b}\left(\sigma_{ax}\right)\cdot C}=\varepsilon_{app}\left(\sigma_{ax}\right)+\frac{V_{S,0}\cdot \sigma_{ax}}{V_{b}\left(\sigma_{ax}\right)\cdot C}$$

*C* describes mainly the elastic compressibility of the solid under uniaxial compression and is associated with the applied determination method. Therefore, *C* is not a directly measured material property and cannot be equated with the bulk modulus of single crystals. The solid compressibility term can be included in common compression equations, such as the model of Heckel and enable the determination of specific compression parameters under the consideration of solid compressibility.

### 5.4. Extension of the Model of Heckel by the Solid Compressibility Term

For the application of the solid compressibility term, the Heckel model (Equation (1)) is transformed into its exponential form:

(12) $$\varepsilon_{app}\left(\sigma_{ax}\right)=e^{\left(-\frac{\sigma_{ax}}{P_{y}}-A\right)}$$

The limit value of the exponential term is zero and this is the necessary condition for the determination of the elastic compressibility factor *C*, which is determined by fitting of Equations (11) and (12) to the apparent in-die porosity curve from compaction experiments. Further details of the fitting procedure are summarized in chapter 4.4.

The elastic compressibility factor C, determined by the mathematical approach, is comparable with the bulk modulus K characterized by mercury porosimetry for Para and MCC (Table 4). The elastic compressibility factor of Lac is somewhat lower compared to the bulk modulus, so that the influence of solid compressibility is slightly overestimated. One possible reason for the deviation is the mathematical method for the determination of C, which is strongly dependent on the applied models. Additionally, the transferability of the bulk modulus determined by hydrostatic compression with a defined pressure to uniaxial powder compression with an unknown and complex stress distribution on the single particles might be limited. Moreover, the back-calculated elastic compressibility factor is also influenced by other mechanisms like die expansion. For DCPA, C is considerably larger than the values for the other three materials which correlates well with the expectation based on mercury porosimetry. The correlation of the elastic compressibility factor with the bulk modulus is generally good and confirms the suitability of the introduced mathematical approach for the consideration of the effect of solid compressibility. The introduced approach provides an inexpensive method for the correction of negative apparent in-die porosities and thus for the convergence of the apparent in-die porosity to the real in-die porosity, which is not directly accessible.

**Figure 5:**
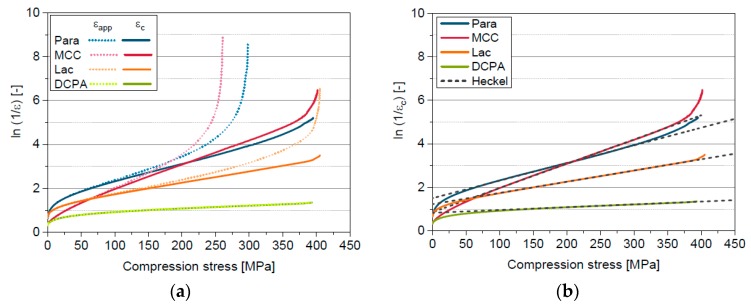
(**a**) Heckel plots calculated using the apparent and the corrected in-die porosity; (**b**) corrected Heckel plots and applied model of Heckel for Para, MCC, Lac and DCPA.

The consideration of C for the calculation of in-die porosities leads to a considerable shift of the Heckel plot to lower values for Para, MCC and Lac (Figure 5). The linear region and by that the applicability of the plot in general, is extended to higher compression stresses, which enables the application of the Heckel model to a more suitable fit range When applying the correction the whole pressure range can be used for the evaluation of Para and MCC data. However, the curves are still bent at maximum compression stress due to the still very low porosities above approximately 350 MPa. The real in-die porosity at high compression stresses is expected to be slightly higher but is not experimentally accessible. Additionally, the slopes of the plots become smaller and this results in higher mean yield pressures (Table 5). However, the difference of *P*_y_ and *P*_y,c_ is very small for DCPA because of the high elastic compressibility factor. The differentiation of the compression behaviour of the different materials by *P*_y,c_ is successful. The finding of the highest mean yield pressure for DCPA, the lowest for MCC and a medium value for Lac correlates well with the structural observation (Section 5.1) and with literature [23,47,48]. Commonly, the yield pressure is used for the classification of the plasticity of materials. It is assumed that low mean yield pressures, which indicate a low resistance against compression, are an indicator for a high plasticity, while the plasticity is decreasing with increasing mean yield pressure [47]. According to the derived mean yield pressures, MCC and Para possess the highest plasticity followed by Lac, while DCPA has the lowest plasticity overall (Table 5).

It can be concluded that the quantification and differentiation of the resistance of the powder against compression by Heckel analysis is successful. However, the Heckel model describes only the linear region of the Heckel plot and not the entire compression curve. Deviations are visible in the low and high compression stress range, which are also the reason for the overall low goodness-of-fit, if the whole compression curve is considered. The comprehensive differentiation of the compressibility requires the description of the entire in-die compression curves. A model, which is better suited for the description of the entire curve is the model of Cooper & Eaton [16].

### 5.5. Extension of the Model of Cooper & Eaton by the Solid Compressibility Term

**Figure 6:**
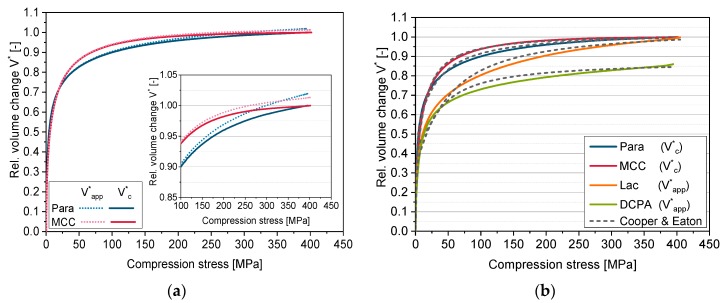
(**a**) Apparent and corrected relative volume change for Para and MCC; (**b**) Relative volume change and applied model of Cooper & Eaton for Para, MCC, Lac and DCPA.

The criterion for the correction by the solid compressibility term (Equation (11)) when applying the model of Cooper & Eaton is to keep the relative volume change in the considered compression stress range (up to 400 MPa) lower than the limiting value of one. The model of Cooper & Eaton consists of two exponential terms, which are, however, able to describe relative volume changes above one. Therefore, the fitting of the model including the solid compressibility term was performed using the constraint: V* ≤ 1. Thus, in the considered case, the solid compressibility term is only applicable if the apparent relative volume change converges to values above one, which is only the case for MCC and Para (Figure 6). The derived elastic compressibility factors C for Para and MCC are comparable to the values derived by the model of Heckel and are thus as well in good accordance with the bulk modulus *K* (Table 4).

**Figure 7:**
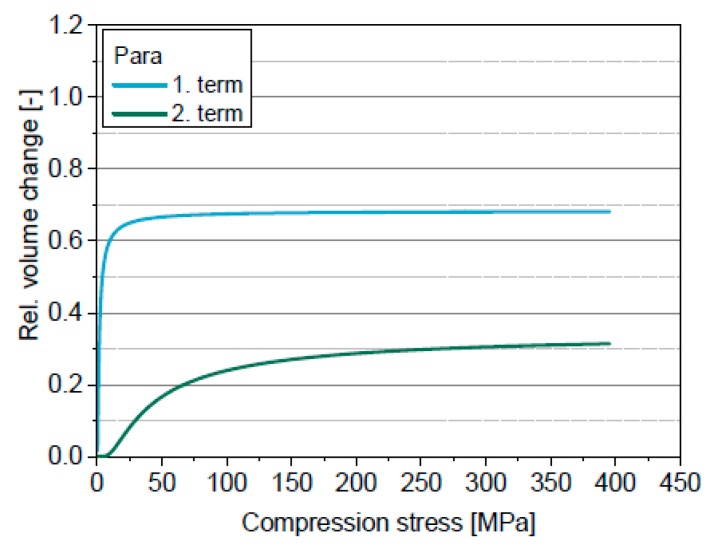
Individual terms of the model of Cooper & Eaton for Para.

The four-parametric function of Cooper & Eaton enables the mathematical description of the whole in-die compression curve. The deviations between experimental data and the model are small for all four powders (Figure 6) and thus the correlation coefficients are high (Table 6). Obviously, the first term mainly describes the volume change at low compression stresses (<25 MPa), while the second term is almost zero up to 10 MPa and primarily describes the volume change at higher compression stresses (Figure 7).

The influence of the solid compressibility term on the derived specific compression parameters is very small (Table 6). Only *k*_2_ of Para clearly decreases. *k*_1_, significant in the low compression stress range, is the lowest for DCPA followed by Lac, MCC and Para. The resistance against filling of large pores thus increases in this order. However, the differences between the four materials are small. *k*_2_ differs more clearly. In this stress range, MCC shows the lowest resistance against the filling of small pores by deformation followed by DCPA, Para and Lac. The lowest compression resistance was expected for MCC and the highest for DCPA based on the slopes of the compression curves. Furthermore, the inapplicability of the solid compressibility term for Lac and DCPA has to be taken into account. The differentiation of the materials based on the resistance values of the model of Cooper & Eaton is difficult as the parameters cannot be correlated to macroscopically reasonable rank orders of the materials, as found as well by Sonnergaard [49]. Therefore, an extended in-die compression function is developed, which is based on the models of Heckel and of Cooper & Eaton.

### 5.6. Extended in-die Compression Function

The extended in-die compression function consists also of two exponential terms (Equation (13)) besides the solid compressibility term (Equation (11)). The counter and denominator of the exponent of the exponential terms are inverted compared with the model of Cooper & Eaton and, by that, in accordance to the model of Heckel. The limit value of these terms is zero, so that the solid compressibility term is applicable in combination with this function.

(13) $$\varepsilon_{c,fit}\left(\sigma_{ax}\right)=\varepsilon_{l}\cdot exp\left(-\frac{\sigma_{ax}}{\sigma_{l}}\right)\;+\;\varepsilon_{h}\cdot exp\left(-\frac{\sigma_{ax}}{\sigma_{h}}\right)$$

where *ε*l is the porosity change attributed to the low stress process and *σ*_l_ is the bulk densification strength which is significant for the low stress process, while *ε*h is the porosity change attributed to the high stress process and *σ*_h_ is the material densification strength of the high stress process. The sum of *ε*l and *ε*h equals the initial porosity at 0 MPa.

**Figure 8:**
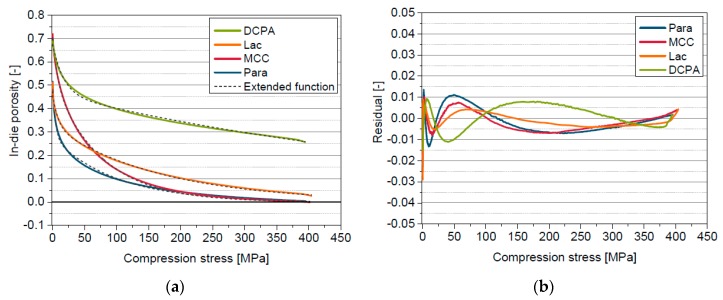
(**a**) Corrected in-die porosity curves and applied extended compression function; (**b**) Residual plot for the fitted extended compression function for Para, MCC, Lac and DCPA.

The in-die porosity curves are shifted or rather bent into the positive porosity range compared with the apparent in-die porosity curves (Figure 8), by mathematically considering solid compressibility. The derived in-die porosity approaches zero for materials with low densification strength. The curves of all four materials are bent to lower porosities just before reaching the maximum compression stress. This curvature at maximum compression stress is technically caused but does not represent the behaviour of the powder and therefore can be considered as measurement artefact due to construction, instrumentation and operation of the compaction simulator. Based on the small stress range in which this curvature occurs, it is not significant for the application of the extended in-die compression function. The deviations between in-die porosity and the function are small (Figure 8) and the correlation coefficients (Table 7) are very high for all investigated materials. The derived elastic compressibility factors are in good accordance with the bulk moduli for MCC and Para and are comparable to the values derived by applying the solid compressibility term in combination with the model of Heckel (Table 4), except for DCPA. The elastic compressibility factor C of DCPA, derived by the extended in-die compression function, is considerably higher. The in-die compression curve of DCPA does approximate neither the zero line nor a plateau value in the applied compression stress range. The in-die porosities at maximum compression stress are still very high (>20%). However, one criterion of the determination of C is the convergence of the curve to the zero line. This range is clearly outside (>1000 MPa) the considered compression stress range which in turn leads to the mathematically uncertain determination of C. Furthermore, a deviation between C and K is visible for Lac, which was already discussed in Chapter 5.4. 

**Figure 9:**
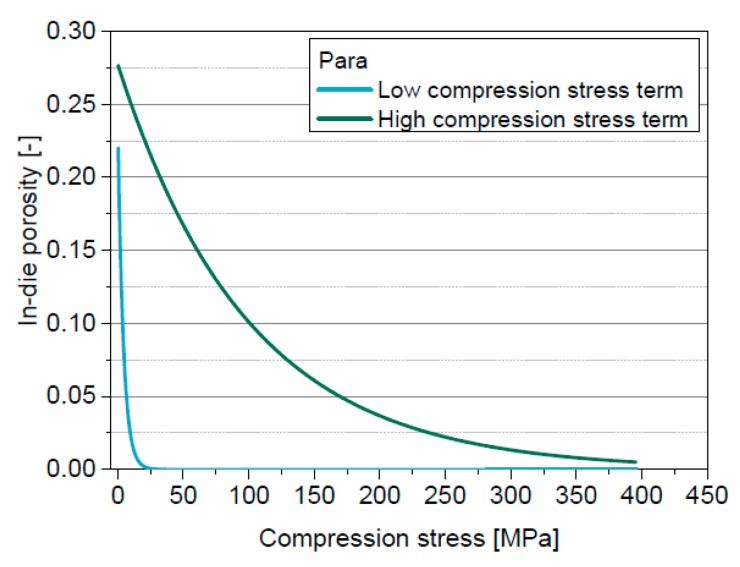
Individual terms of the extended in-die compression function for Para.

The first exponential term describes the low compression stress range, while the second exponential term mainly describes the high compression stress range (Figure 9). A clear distinction of the bulk densification strength *σ*_l_ of the four materials in the low compression stress range is visible (Table 7). *σ*_l_ is most likely mainly dependent on the friction between particles and their Young’s modulus. *σ*_l_ rises in the order Para < MCC < Lac < DCPA. This correlates well with the experimental findings. The lowest bulk densification strength for Para may be explained by a facilitated particle rearrangement due to the rectangular particle shape. *σ*_l_ of MCC is also low because of a facilitated particle rearrangement due to the initially loose packing (initial porosity > 0.7) of the elongated particles. The particle rearrangement of Lac may be hindered due to a closer initial packing of the particles because of the very broad particle size distribution. The reasons for the highest *σ*_l_ for DCPA might be the hindered particle rearrangement due to a probably already very close packing of the spherical particles and the low elasticity. These are certainly only hypothetical considerations, which have to be proved by further investigations.

The material densification strength *σ*_h_ is the lowest for MCC. Para shows a slightly higher resistance against compression followed by Lac. DCPA has the overall highest material densification strength. The material densification strength of Para and MCC can be explained by the mainly elastic and plastic deformation behaviour of these materials. The medium material densification strength of Lac may be traced back to the higher necessary compression stress for the breakage of the agglomerated/aggregated particles and the higher resistance of the primary particles against deformation due to their smaller particle size. The reasons for the considerably higher material densification strength of DCPA might be the high stiffness and low elasticity of this material.

The extended in-die compression function is well-suited for the precise description of the in-die compression curves of all model materials and successfully mathematically considers the physical effect of solid compressibility. Additionally, the extended in-die compression function enables the successful quantification and differentiation of the compression behaviour of the materials investigated.

### 5.7. Comparison in- and out-of-die Compressibility

**Figure 10:**
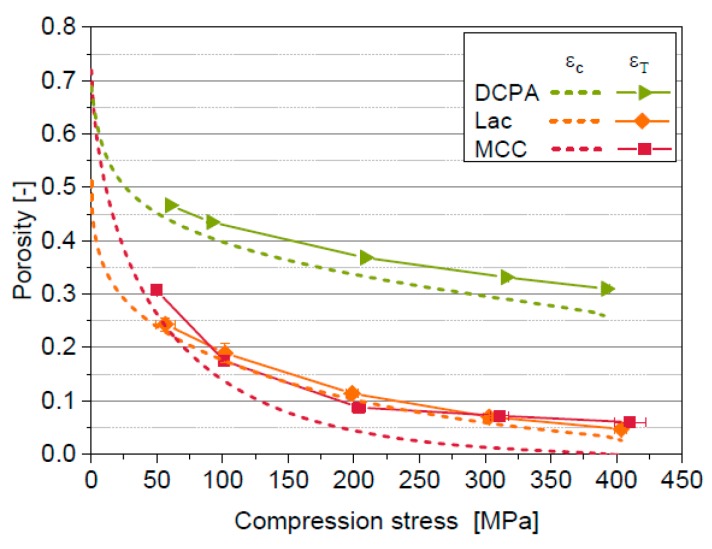
Comparison of in-die porosity considering solid compressibility *ε*_c_ with out-of-die compressibility *ε*_T_ for DCPA, Lac and MCC.

Solid compressibility is only one part of the overall elastic deformation during powder compression. Another factor is the elastic deformation and relaxation of the framework of the particles, which is not considered by the introduced mathematical approach yet because of considering only the compression curve but not the decompression curve so far. The elastic deformation of the framework is thus the main reason for the differences between the in-die compressibilities considering solid compressibility *ε*_c_ and the out-of-die compressibilities *ε*_T_ (Figure 10). Therefore, the remaining differences provide information on the elastic deformation of the framework within the bulk. It can be deduced that the elastic relaxation of the bulk framework of Lac is least pronounced while the highest values are measured for MCC. It needs to be clarified whether this can be described by an intercorrelation between solid compressibility and deformation mechanisms of the material. Therefore, the consideration of the decompression curve is necessary. The out-of-die compressibility of Para cannot be determined because of the occurrence of tablet defects due to capping. The differences of the compressibility curves also cause differences of the mean yield pressures derived by in-die Heckel analysis considering solid compressibility *P*_y,c_ compared to out-of-die Heckel analysis *P*_y,T_ (Table 5). *P*_y,c_ is lower compared to *P*_y,T_ because of the consideration of the elastic deformation of the particle framework. The deformation behaviour of MCC is classified as brittle based on *P*_y,T_. This is not reasonable because of the evidently plastic deformation behaviour of MCC [23,48]. The high mean yield pressure *P*_y,T_ can be traced back to the small slope of the out-of-die compressibility between 200 MPa and 400 MPa. Furthermore, the mean yield pressure is dependent on the considered compression stress range. The compression behaviour of a powder is not only affected by plastic deformation and particle fragmentation but rather by all acting micro-processes including particle rearrangement and elastic deformation. Therefore, the comprehensive characterization of the compression behaviour requires the consideration of the entire compression curves including the effect of all these micro-processes. The introduced in-die approach enables such a comprehensive characterization of powder compressibility even if tablet defects occur.

The estimation of the complete elastic recovery of the bulk solely based on in-die compression by a further development of the presented method using the decompression curve is a prospective aim.

## 6. Conclusions

The physical effect of solid compressibility, which causes the increase of solid density with rising compression stress, cannot be neglected for the calculation of in-die porosities. The experimental determination of the bulk modulus is difficult and expensive. Therefore, a mathematical term for the consideration of solid compressibility is introduced based on physical considerations. This term can be used for the extension of common mathematical models, such as the models of Heckel and of Cooper & Eaton providing an inexpensive method for the consideration of the influence of solid compressibility during uniaxial powder compression. This method can be applied even if tablet defects occur. Additionally, an extended in-die compression function is introduced based on the common models of Heckel and of Cooper & Eaton. This function precisely describes the entire in-die compression curves and enables the successful differentiation and quantification of the compression behaviour of the investigated pharmaceutical powders.

The consideration of solid compressibility leads to the approach of the in-die compression curves to the out-of-die compression curves. However, differences are still visible because of the elastic recovery of the bulk, which is not considered yet. The estimation of the elastic behaviour of the bulk solely based on in-die compression by consideration of the decompression curve and thus the further development of the presented method, is a prospective aim. Continuing, the systematic investigation of the influence of material parameters (e.g., deformation behaviour, particle size and morphology) up to complex mixtures is required to allow the evaluation of the proposed model for realistic formulations.

## Figures and Tables

**Table 1:** Characteristic particle sizes and solid density.

Material	*x*_10_ [µm]	*x*_50_ [µm]	*x*_90_ [µm]	*ρ*_s_ [g/cm^3^]
MCC	36	125	261	1.5537 ± 0.0080
Para	4	25	72	1.2863 ± 0.0001
Lac	12	147	342	1.5524 ± 0.0077
DCPA	77	190	302	2.7718 ± 0.0097

**Table 2:** Bulk modulus determined by mercury porosimetry and minimal in-die porosities.

Material	Bulk Modulus *K* [GPa]	Min. Apparent in-die porosity ε_app,He,min_ [-]	Min. in-die porosity ε_c,Hg,min_ [-]
MCC	10.22 ± 1.02	−0.035	0.006
Para	14.93 ± 3.67	−0.023	0.005
Lac	27.07 ± 7.13	0.001	0.016
DCPA	-	0.256	-

**Table 3:** Stress ratio, die expansion coefficient and porosity change due to die expansion (*n* = 6).

Material	Stress Ratio [-]	Die Expansion Coefficient [GPa]	Difference of Porosity at 400 MPa [-]
MCC	0.47–0.81	207 ± 4	3.7 × 10^−6^
Para	0.50–0.85	196 ± 3	4.2 × 10^−6^
Lac	0.45–0.71	237 ± 4	2.9 × 10^−6^
DCPA	0.21–0.43	382 ± 5	1.1 × 10^−6^

**Table 4:** Bulk modulus determined by mercury porosimetry and elastic compressibility factors derived by extension of the models of Heckel and of Cooper & Eaton with the solid compressibility term and by the extended in-die compression function.

Material	Bulk Modulus K [GPa]	*C*_Heckel_ [GPa]	*C*_Cooper &Eaton_ [GPa]	*C*_Extended function_ [GPa]
MCC	10.22 ± 1.02	11.51 ± 0.17	11.87 ± 0.12	11.74 ± 0.30
Para	14.93 ± 3.67	15.22 ± 0.07	18.41 ± 0.74	15.97 ± 1.00
Lac	27.07 ± 7.13	14.09 ± 0.03	-	14.80 ± 1.46
DCPA	-	150.02 ± 17.24	-	277.06 ± 10.02

**Table 5:** Parameters derived by Heckel analysis (*n* = 6).

Material	*P*_y_ [MPa]	Fit Range	With Solid Compressibility Term			*P*_y,T_ [MPa]
			*P*_y,c_ [MPa]	Fit Range	*R*^2^ [-] Entire Curve	
MCC	72.6 ± 1.0	10–50% *σ*_max_	89.3 ± 1.2	40–90% *σ*_max_	0.9860	543.5
Para	93.5 ± 1.2	6–56% *σ*_max_	121.2 ± 2.7	30–80% *σ*_max_	0.9739	-
Lac	145.6 ± 0.5	25–75% *σ*_max_	194.5 ± 0.6	40–90% *σ*_max_	0.9935	233.1
DCPA	765.4 ± 1.5	38–88% *σ*_max_	771.2 ± 2.5	40–90% *σ*_max_	0.9313	1062.6

**Table 6:** Parameters derived by the fitting of the model of Cooper & Eaton (*n* = 6).

Material	V^*^_app_				With Solid Compressibility Term V^*^_c_				
	*a*_1_ [-]	*k*_1_ [MPa]	*a*_2_ [-]	*k*_2_ [MPa]	*a*_1c_ [-]	*k*_1c_ [MPa]	*a*_2c_ [-]	*k*_2c_ [MPa]	*R*^2^ [-]
MCC	0.58 ± 0.01	1.46 ± 0.16	0.45 ± 0.01	20.2 ± 1.0	0.57 ± 0.01	1.43 ± 0.20	0.45 ± 0.01	18.7 ± 0.9	0.9998
Para	0.70 ± 0.003	1.40 ± 0.10	0.35 ± 0.003	43.7 ± 2.4	0.69 ± 0.003	1.37 ± 0.10	0.34 ± 0.003	38 ± 2.0	0.9993
Lac	0.52 ± 0.002	0.84 ± 0.00	0.54 ± 0.002	53.6 ± 0.4					0.9963
DCPA	0.45 ± 0.01	0.96 ± 0.10	0.42 ± 0.009	29.2 ± 1.9					0.9926

**Table 7:** Specific compression parameters of the extended in-die compression function (*n* = 6).

Material	*ε*_l_ [-]	*σ*_l_ [MPa]	*ε*_h_ [-]	*σ*_h_ [MPa]	*C* [GPa]	*R*^2^ [-]
MCC	0.20 ± 0.00	8.5 ± 0.6	0.51 ± 0.01	79.0 ± 1.9	11.74 ± 0.30	0.9998
Para	0.24 ± 0.00	4.3 ± 0.1	0.28 ± 0.00	99.2 ± 3.2	15.97 ± 1.00	0.9993
Lac	0.14 ± 0.01	9.4 ± 0.5	0.32 ± 0.00	177.3 ± 5.6	14.80 ± 1.46	0.9997
DCPA	0.21 ± 0.00	16.8 ± 0.2	0.47 ± 0.00	668.6 ± 3.5	277.06 ± 10.20	0.9993

## References

[1] Podczeck F., Sharma M. (1996). The influence of particle size and shape of components of binary powder mixtures on the maximum volume reduction due to packing. Int. J. Pharm..

[2] Tye C.K., Sun C., Amidon G.E. (2005). Evaluation of the effects of tableting speed on the relationships between compaction pressure, tablet tensile strength, and tablet solid fraction. J. Pharm. Sci..

[3] Patel S., Kaushal A.M., Bansal A.K. (2006). Effect of Particle Size and Compression Force on Compaction Behavior and Derived Mathematical Parameters of Compressibility. Pharm. Res..

[4] Kaerger J., Edge S., Price R. (2004). Influence of particle size and shape on flowability and compactibility of binary mixtures of paracetamol and microcrystalline cellulose. Eur. J. Pharm. Sci..

[5] David S.T., Augsburger L.L. (1977). Plastic Flow during Compression of Directly Compressible Fillers and Its Effect on Tablet Strength. J. Pharm. Sci..

[6] Hiestand E.N., Wells J.E., Peot C.B., Ochs J.F. (1977). Physical Processes of Tableting. J. Pharm. Sci..

[7] Leuenberger H., Rohera B.D. (1986). Fundamentals of Powder Compression. I. The Compactibility and Compressibility of Pharmaceutical Powders. Pharm. Res..

[8] USP (2017). Tablet Compression Characterization, Second Supplement to USP 40-NF 35.

[9] Train D. (1956). An investigation into the compaction of powders. J. Pharm. Pharmacol..

[10] Wray P.E. (2008). The Physics of Tablet Compaction Revisited. Drug Dev. Ind. Pharm..

[11] Hiestand E.N. (1966). Powders: Particle-Particle Interactions. J. Pharm. Sci..

[12] Hiestand E.N. (1985). Dispersion Forces and Plastic Deformation in Tablet Bond. J. Pharm. Sci..

[13] Kawakita K., Lüdde K.-H. (1971). Some considerations on powder compression equations. Powder Technol..

[14] Çelik M. (2008). Overview of Compaction Data Analysis Techniques. Drug Dev. Ind. Pharm..

[15] Heckel R.W. (1961). Density-Pressure Relationships in Powder Compaction. Trans. Metall. AIME.

[16] Cooper A.R., Eaton L.E. (1962). Compaction Behavior of Several Ceramic Powders. J Am. Ceram. Soc..

[17] Sun C., Grant D.J. (2001). Influence of Elastic Deformation of Particles on Heckel Analysis. Pharm. Dev. Technol..

[18] Katz J.M., Buckner I.S. (2017). Full Out-of-Die Compressibility and Compactibility Profiles From Two Tablets. J. Pharm. Sci..

[19] Sonnergaard J. (1999). A critical evaluation of the Heckel equation. Int. J. Pharm..

[20] Katz J.M., Roopwani R., Buckner I.S. (2013). A Material-Sparing Method for Assessment of Powder Deformation Characteristics Using Data Collected During a Single Compression–Decompression Cycle. J. Pharm. Sci..

[21] Pederson S., Kristensen H.G. (1971). Compressibility of 18 Molecular Organic Solids to 45 kbar. J. Chem. Phys..

[22] Klevan I., Nordström J., Bauer-Brandl A., Alderborn G. (2009). On the physical interpretation of the initial bending of a Shapiro–Konopicky–Heckel compression profile. Eur. J. Pharm. Biopharm..

[23] Patel S., Kaushal A.M., Bansal A.K. (2010). Mechanistic investigation on pressure dependency of Heckel parameter. Int. J. Pharm..

[24] Boldyreva E.V. (2008). High-pressure diffraction studies of molecular organic solids. A personal view. Acta Crystallogr. A Found. Crystallogr..

[25] Fabbiani F.P.A., Allan D.R., David W.I.F., Davidson A.J., Lennie A.R., Parsons S., Pulham C.R., Warren J.E. (2007). High-Pressure Studies of Pharmaceuticals: An Exploration of the Behavior of Piracetam. Cryst. Growth Des..

[26] Vaidya S.N., Kennedy G.C. (1971). Compressibility of 18 Molecular Organic Solids to 45 kbar. J. Chem. Phys..

[27] Gane P.A., Kettle J.P., Matthews P.G., Ridgway C.J. (1996). Void Space Structure of Compressible Polymer Spheres and Consolidated Calcium Carbonate Paper-Coating Formulations. Ind. Eng. Chem. Res..

[28] Ridgway C.J., Ridgway K., Matthews P.G. (1997). Modelling of the Void Space of Tablets Compacted Over a Range of Pressures. J. Pharm. Pharmacol..

[29] Sun C.C. (2008). Mechanism of moisture induced variations in true density and compaction properties of microcrystalline cellulose. Int. J. Pharm..

[30] Gabaude C.M., Guillot M., Gautier J., Saudemon P., Chulia D. (1999). Effects of true density, compacted mass, compression speed, and punch deformation on the mean yield pressure. J. Pharm. Sci..

[31] Sun C. (2005). True Density of Microcrystalline Cellulose. J. Pharm. Sci..

[32] Heckel R.W. (1962). A Normalized Density-Pressure Curve for Powder Compaction. Trans. Metall. AIME.

[33] Hersey J.A., Rees T.E. (1970). Proceeding of the Second Particle Size Analysis Conference.

[34] Beitz W., Grote K.-H. (1997). Dubbel Taschenbuch für den Maschinenbau.

[35] Perry J., Aboudi J. (2003). Elasto-Plastic Stresses in Thick Walled Cylinders. Trans. ASME.

[36] Sun C. (2004). A Novel Method for Deriving True Density of Pharmaceutical Solids Including Hydrates and Water-Containing Powders. J. Pharm. Sci..

[37] Viana M. (2002). About pycnometric density measurements. Talanta.

[38] Mazel V., Busignies V., Diarra H., Tchoreloff P. (2012). Measurements of Elastic Moduli of Pharmaceutical Compacts: A New Methodology Using Double Compaction on a Compaction Simulator. J. Pharm. Sci..

[39] Mazel V., Busignies V., Diarra H., Tchoreloff P. (2013). On the Links Between Elastic Constants and Effective Elastic Behavior of Pharmaceutical Compacts: Importance of Poisson’s Ratio and Use of Bulk Modulus. J. Pharm. Sci..

[40] Bassam F., York P., Rowe R.C., Roberts R.J. (1990). Young’s modulus of powders used as pharmaceutical excipients. Int. J. Pharm..

[41] Higuchi T., Shimamoto T., Eriksen S.P., Yashiki T. (1965). Physics of Tablet Compression XIV. Lateral Die Wall Pressure During and After Compression. J. Pharm. Sci..

[42] Windheuser J.J., Misra J., Eriksen S.P., Higuchi T. (1963). Physics of Tablet Compression XIII. J. Pharm. Sci..

[43] Abdel-Hamid S., Betz G. (2010). Study of radial die-wall pressure changes during pharmaceutical powder compaction. Drug Dev. Ind. Pharm..

[44] Brewin P.R. (2008). Modelling of Powder Die Compaction.

[45] Abdel-Hamid S. (2011). Instrumentation of Tableting Machines: High Speed Compaction Investigation through Simulation and Radial Die-Wall Pressure Monitoring. Ph.D. Thesis.

[46] Murnaghan F.D. (1944). The Compressibility of Media under Extreme Pressures. Proc. Natl. Acad. Sci. USA.

[47] Zhang Y., Law Y., Chakrabarti S. (2003). Physical properties and compact analysis of commonly used direct compression binders. AAPS PharmSciTech.

[48] Haware R.V., Tho I., Bauer-Brandl A. (2009). Application of multivariate methods to compression behavior evaluation of directly compressible materials. Eur. J. Pharm. Biopharm..

[49] Sonnergaard J. (2001). Investigation of a new mathematical model for compression of pharmaceutical powders. Eur. J. Pharm. Sci..

